# Genetic Variants in the ATF6 Gene and Their Relationship with Milk-Quality Traits in Yaks

**DOI:** 10.3390/ani15172524

**Published:** 2025-08-27

**Authors:** Xiaoming Ma, Xian Guo, Yongfu La, Xiaoyun Wu, Min Chu, Pengjia Bao, Ping Yan, Chunnian Liang

**Affiliations:** 1 Animal Science Department, Lanzhou Institute of Husbandry and Pharmaceutical Sciences, Chinese Academy of Agricultural Sciences, Lanzhou 730050, China; maxiaoming@caas.cn (X.M.); guoxian@caas.cn (X.G.); layongfu@caas.cn (Y.L.); wuxiaoyun@caas.cn (X.W.); chumin@caas.cn (M.C.); baopengjia@caas.cn (P.B.); 2 Key Laboratory of Animal Genetics and Breeding on Tibetan Plateau, Ministry of Agriculture and Rural Affairs, Chinese Academy of Agricultural Sciences, Lanzhou 730050, China; 3 Key Laboratory for Yak Genetics, Breeding, and Reproduction Engineering of Gansu Province, Lanzhou 730050, China; 4 Institute of Western Agriculture, Chinese Academy of Agricultural Sciences, Changji 931100, China

**Keywords:** yak, ATF6, SNP, milk quality

## Abstract

Yaks (*Bos grunniens*) are a predominant livestock species on the Tibetan Plateau, known for their adaptability to the cold and dry climate typical of this region. This study investigates the association of two SNPs within the ATF6 gene (Chr3:9812652G>T (CM016692.1) and Chr3:9900243C>T (CM016692.1)) with key milk-quality traits in yaks. Due to the low frequency of TT homozygotes (<5%), analysis focused on major genotypes: GG vs. GT and CC vs. CT. Results from the general linear models revealed that the g.3_9812652G>T variant was significantly associated with increased levels of casein, protein, acidity, and solid-not-fat (SNF) in GT individuals (*p* < 0.01). No significant differences were observed for lactose, urea, citric acid, or fat. For g.3_9900243C>T, CT individuals showed higher casein, protein, SNF, and citric acid levels compared to CC (*p* < 0.05). These results suggest both SNPs are linked to key milk traits, especially protein, casein, and SNF. The g.3_9812652G>T variant had a stronger and more consistent effect, indicating it may play a larger role in milk composition regulation. Overall, ATF6 is a promising candidate gene for marker-assisted selection (MAS) to improve milk quality in yaks. Further studies in larger and more diverse populations are needed to confirm these findings and explore the gene’s functional role.

## Simple Summary

Yaks (*Bos grunniens*) are the most important type of livestock on the Tibetan Plateau, providing milk, meat, and fiber for herders living in extreme high-altitude conditions. Improving milk quality is essential for both human nutrition and sustainable pastoral livelihoods. In this study, we focused on the ATF6 gene, a key regulator of protein folding and secretion in mammary cells, and examined its genetic variation in 291 lactating yaks using a targeted sequencing platform. By linking genetic markers with milk-quality traits, this work highlights the potential of ATF6 as a candidate gene for marker-assisted selection, offering a molecular basis for breeding programs aimed at enhancing the nutritional value and economic importance of yak milk.

## 1. Introduction

The yak (*Bos grunniens*) is a unique high-altitude domestic species that is primarily found in the Tibetan Plateau and the Himalayas; the species exhibits exceptional adaptability to cold and hypoxic environments thanks to features such as persistent fetal hemoglobin, enlarged cardiopulmonary capacity, and thick subcutaneous fat, enabling survival at altitudes ranging from 2000 to 6000 m a.s.l [1]. Yak milk is rich in bioactive components such as immunoglobulins and lactoferrin, which contribute to its nutritional and health-promoting value [2,3,4]. Yaks are essential to the livelihoods of herders, providing meat, milk, fiber, draft power, and dried dung as a major fuel source. Among these, yak milk is a highly valued product that is rich in nutrients and known for its bioactive properties [5]. Yak milk has a high nutritional content, with approximately 4.0–5.9% protein, 5.5–7.5% fat, and 4.0–5.9% lactose, along with abundant minerals and vitamins (e.g., calcium, magnesium, phosphorus). Its fatty acid profile is dominated by saturated fats (65–75%), with significant proportions of monounsaturated (20–25%) and polyunsaturated acids (3–6%) [3]. Functional studies, especially on casein-derived peptides from yak milk, have demonstrated multiple health-promoting activities, including antioxidant, antihypertensive (through ACE inhibition), antibacterial, anti-inflammatory, anticancer, and immunomodulatory effects [6,7]. These findings support the potential of yak milk and its bioactive components for development into functional dairy products. Traditionally, yak milk is consumed fresh or processed into butter, yogurt, cheese, and Tibetan-style milk tea products, such as “chhurpi” hard cheese and plateau butter, which serve as daily dietary staples and marketable commodities, highlighting the importance of yak milk in ensuring nutrition and supporting sustainable development in high-altitude pastoral systems [3].

With increasing demand for higher protein content and overall quality in milk, traditional breeding methods—which often achieve genetic progress slowly and with limited efficiency—are increasingly being supplemented by integrated molecular breeding strategies. Gene discovery tools such as genome-wide association studies (GWAS), SNP mapping, and capture-based targeted sequencing (cGPS) are widely used to identify genomic regions of interest. These discoveries, in turn, enable the application of marker-assisted selection (MAS) and other precision breeding techniques. Single-nucleotide polymorphisms (SNPs), as third-generation molecular markers, offer high resolution and broad applicability. Bovine genomes contain roughly 80 million SNPs, while humans have 3–10 million, averaging one SNP per kilobase. These advances have enabled the identification of genes affecting milk yield, fat, and protein traits. Notably, Ma et al. genotyped 172 Gannan yaks using cGPS and identified three novel SNPs in *PRKD1* (g.283619T>C, g.283659C>A) and *KCNQ3* (g.133741T>C), which were significantly correlated with increased lactose, fat, casein, protein, SNF (non-fat milk solids), and acidity in yak milk [8]. Similarly, Feng et al. detected three SNPs in *PRKG1*—g.404195C>T, g.404213C>T, and g.760138T>C—in 172 Gannan yaks; specific genotypes (e.g., TT at g.404195C>T) were associated with significantly higher casein, total protein, and SNF levels [9]. Moreover, in Chinese Holstein cattle, Shi et al. linked *PRKG1* SNPs to milk fatty acids such as C8:0, C10:0, C16:1, and C17:1 (*p* < 0.0001–0.0123), demonstrating that variants can alter transcription and enzymatic lipolysis in adipocytes and suggesting that genetic variants may contribute to differences in fatty acid composition [10]. These findings align with earlier studies in *Bos taurus* cattle that identified genes such as *DGAT1*, *SCD1*, *FAT3*, *HTR1B*, *CPM*, *MINPP1*, and *LIPJ* as influential for milk production and composition traits [11]. PRKG1 spans about 1.44 Mb, with 20 exons, and encodes a cGMP-dependent protein kinase involved in regulating lipid metabolism, feed efficiency, and milk composition traits in cattle. Recent studies in Chinese Holstein cattle have linked *PRKG1* SNPs to variation in milk fatty acid content [10]. Therefore, *PRKG1* and other candidate genes (*PRKD1*, *KCNQ3*, *DGAT1*, *GHR*) may provide useful genomic markers for improving milk protein and fat traits in yaks. In summary, these genetic findings lay a solid groundwork for future precision breeding programs to enhance milk yield and composition in yaks and other ruminants.

Activating transcription factor 6 (ATF6) is an ER-stress-sensing transcription factor that regulates the ER folding quality control mechanism in the unfolded protein response (UPR); it plays a particularly important role in high metabolic breast cells for lipid and protein synthesis [12]. ATF6 expression is significantly upregulated in the bovine mammary gland during periods of metabolic and lactational stress—such as the onset of lactation—alongside other UPR signaling factors like *PERK*, *IRE1α*, *GRP78* (*BiP*), and *CHOP*. This pattern suggests ATF6 plays a role in adaptive unfolded-protein response mechanisms that help restore endoplasmic reticulum homeostasis and support mammary epithelial secretory function [13]. However, to date, no GWAS or QTL studies have demonstrated associations between ATF6 genetic variants and milk yield or composition traits, whereas other UPR-related genes such as GRP78 have documented roles in regulating milk biosynthesis and mammary cell proliferation [14]. Therefore, while ATF6 remains a physiologically relevant component of the lactational UPR network, its role as a candidate gene in yak milk trait variation should be framed as provisional, i.e., worthy of future investigation rather than a currently established genetic regulator. These UPR responses are believed to help restore endoplasmic reticulum homeostasis and maintain mammary cell secretion function, thereby indirectly affecting the milk protein content and fatty acid composition. For example, supplementing the diet with rumen-protected or rumen undegradable protein (RUP) can enhance UPR signaling and improve milk protein synthesis capacity [15]. Excessive circulating non-esterified fatty acids (NEFAs), mobilized from body fat reserves, can also activate ATF6 and induce ER stress, thereby affecting milk fat synthesis and secretion.

Although there is currently no direct evidence on the association between *ATF6* gene polymorphism and milk-quality traits in yaks, indirect evidence from dairy cows supports the potential impact of ATF6 on milk quality indicators such as milk protein content, fat content, and cell secretion efficiency by regulating mammary gland ER function and the UPR pathway. Our study employs yaks as an experimental model to explore the genetic polymorphisms of ATF6 and their relationship with milk-quality traits in yaks, aiming to provide valuable insights for improving yak milk quality and developing molecular markers for yak populations.

## 2. Materials and Methods

All manipulations of the live yaks in this study were conducted following the Animal Ethics Procedures and Guidelines of the People’s Republic of China and complied with recognized standards for ethical animal care. The research protocol received approval from the Animal Administration and Ethics Committee of the Lanzhou Institute of Husbandry and Pharmaceutical Sciences, Chinese Academy of Agricultural Sciences (Approval No. 2024-53).

### 2.1. Experimental Animals and Milk Composition Analysis

A total of 291 adult lactating female yaks were selected from the Jiali County Ranch in Nagqu City, located in the Tibet Autonomous Region. The animals had no genetic relationship to avoid inclusion of yaks with the same pedigree. Ear tissue samples were collected and promptly placed in liquid nitrogen for short-term preservation, followed by storage at −80 °C in the laboratory. Genomic DNA was extracted using the MGIEasy Genomic DNA Extraction Kit (V1.0, 48Preps, Shenzhen, China) based on the magnetic bead method, in accordance with the manufacturer’s protocol. All sampled yaks were clinically healthy, exhibited consistent body size and milk production levels, and were maintained on natural grazing pastures without supplementation of concentrate or roughage. Milk was obtained manually from the udders of these 291 yaks (with 2–3 calvings) and analyzed for fat, protein, casein, non-fat solids (SNFs), lactose, urea, and CitrAcid. The composition of milk samples was determined using a MilkoScan™ FT120 milk analyzer (Foss Analytical, Hellerup, Denmark).

### 2.2. Genotyping

Genotyping of the 291 yak individuals was performed using the Yak cGPS (Genotyping by Pinpoint Sequencing of captured targets) 10 K liquid-phase chip platform, co-developed by Smartgenomics Technology Institute (Tianjin, China). The cGPS approach utilizes targeted sequence capture in the liquid phase, offering several advantages such as high-resolution genotyping, excellent stability, rich genetic information output, and robust reproducibility [16]. The probe design was guided by candidate genomic regions identified in our unpublished whole-genome resequencing and genome-wide association study (GWAS) of milk-quality traits in yaks. In this preliminary analysis, we used the rMVP R package (rMVP, version1.4.5) and applied its general linear model (GLM) function to detect significant SNP–trait associations. ATF6 emerged as one of the genes showing strong association signals and was therefore included among the targeted regions for probe design [17]. Custom-designed probes were then used to selectively hybridize and enrich these target regions via liquid-phase hybridization. The enriched DNA fragments were subjected to library preparation and high-throughput sequencing, enabling accurate genotyping of markers within these regions.

Raw sequence data generated from the platform contained adapter contamination and low-quality reads. To improve data integrity, Fastp software (version 0.23.4) [18] was employed for quality control and filtering. To assess potential sample contamination, a subset of 10,000 reads from each sample’s FASTQ file was randomly selected and compared against the NCBI-NT database using the BLASTN tool [19]. Clean reads were then mapped to the reference genome of yak (*Bos grunniens* v3.0, GCA_005887515.1) using the Burrows-Wheeler Aligner (BWA, version 0.7.17) [20]. Variant calling for target loci was performed using the HaplotypeCaller tool provided in the Genome Analysis Toolkit (GATK, version 4.1.5.0). Finally, functional annotation of identified variants was conducted with the snpEff tool (version 5.2) [21], which provided insights into the genomic context and potential biological effects of the detected polymorphisms.

### 2.3. Statistical Analysis

The theoretical heterozygosity, observed heterozygosity, effective number of alleles, polymorphism information content, theoretical genotype frequency, genotype deviation, and Hardy–Weinberg test *p*-value for the *ATF6* gene locus were calculated using the GDICALL online software (http://www.msrcall.com/gdicall.aspx (accessed on 13 August 2024). The association between ATF6 gene polymorphisms and milk quality traits in yak was analyzed using a univariate general linear model (GLM) implemented in Python with the statsmodels package (version 0.14.4), including genotype and lactation number (2 or 3 calvings) as fixed factors. For each trait, least squares means (LSM; marginal means) and their standard errors (SE) were obtained from the fitted model to compare genotypes. Statistical significance was set at *p* < 0.05.

## 3. Results

### 3.1. Alignment to the Reference Genome

**Figure 1:**
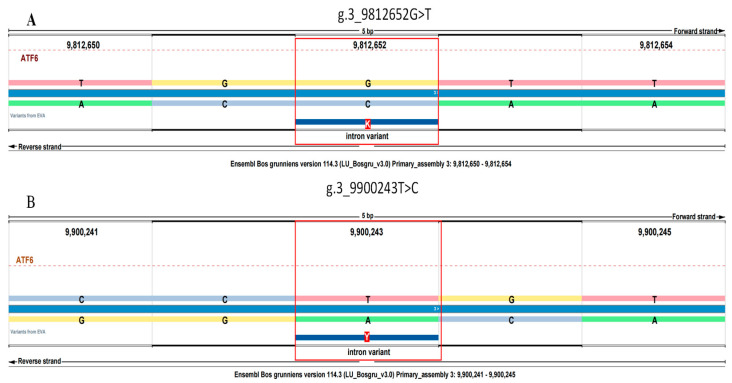
The location of the SNPs in the yak genome. (**A**) g. 3_9812652G > T; (**B**) g. 3_9900243T > C.

Figure 1 illustrates the location of the SNPs within the *ATF6* gene of the yak. The data show that these SNPs exhibit a relatively balanced distribution. Table 1 summarizes the genotype frequencies, allele frequencies, and polymorphism information content (PIC) for two specific SNPs within the *ATF6* gene. The ATF6 g.3_9812652G>T and g.3_9900243T>C variants are located on chromosome 3 at positions 9,812,652 and 9,900,243, respectively, according to the Bos grunniens v3.0 reference genome (GCA_005887515.1) (Figure 1).

The analysis revealed the presence of three genotypes at the two SNP loci in the yak population, specifically ATF6 g.3_9812652G>T and g.3_9900243T>C. Among these, the GG and CC genotypes were the most prevalent, with frequencies of 0.853 and 0.702, respectively. For the g.3_9812652G>T locus, the G allele frequency was 0.921, while the T allele frequency was 0.079, indicating that the G allele is the predominant allele at this intronic site. In contrast, for g.3_9900243T>C, the C allele frequency was 0.827 and the T allele frequency was 0.173, indicating that the C allele is the predominant allele at this intronic site. The PIC value for g.3_9812652G>T was calculated to be 0.135, categorizing this SNP as having low polymorphism, while the PIC value for g.3_9900243T>C was 0.245, classifying it as moderate polymorphism. Moreover, the g.3_9812652G>T SNP follows Hardy–Weinberg equilibrium (*p* > 0.05), while the g.3_9900243T>C SNP does not conform to Hardy–Weinberg equilibrium, with a *p*-value of 0.0312 (*p* < 0.05).

### 3.2. Association Analysis of Milk Traits and Genotypes of SNPs in Yak

In this study, SNP genotyping data from 291 yaks were analyzed to investigate the associations between specific genetic variants and milk composition traits. Table 2 summarizes the results for two SNPs located in the ATF6 gene: g.3_9812652G>T and g.3_9900243C>T. Due to the low frequency of the TT genotype at both loci (less than 5% of the population), only the more prevalent genotypes were included in the statistical analysis. Specifically, comparisons were made between the GG and GT genotypes for g.3_9812652G>T and between the CC and CT genotypes for g.3_9900243C>T. This strategy was adopted to enhance the reliability of the association analysis and reduce the influence of rare genotypes on the overall interpretation of the results.

The association between *ATF6* gene SNPs and milk-quality traits in yak was analyzed using a general linear model (GLM), with genotype and lactation number included as fixed effects. For the g.3_9812652G>T SNP, the GT genotype exhibited significantly higher values for casein (4.53 ± 0.06), protein (5.58 ± 0.10), acidity (13.82 ± 0.26), and SNF (11.74 ± 0.08) compared to the GG genotype. No significant differences were observed for lactose, urea, citric acid, or fat content (*p* > 0.05). Similarly, for the g.3_9900243T>C SNP, CT genotype individuals showed significantly higher levels of casein (4.35 ± 0.04), protein (5.32 ± 0.07), and SNF (11.54 ± 0.06) compared to the CC genotype (*p* < 0.05) and a significant difference in citric acid (*p* = 0.016 in ANOVA, *p* = 0.018 in GLM). However, no significant differences were found for lactose, urea concentration, or fat content (*p* > 0.05). These results confirm that both g.3_9812652G>T and g.3_9900243T>C SNPs are significantly associated with important milk quality traits. Notably, the g.3_9812652G>T SNP demonstrated a stronger and more consistent impact across traits compared to g.3_9900243T>C.

## 4. Discussion

Yak milk is well known for its rich nutritional composition, with well-established compositional ranges reported across breeds such as Maiwa and Gannan yak. In the present study, the overall mean values for milk traits were as follows (mean ± SD): casein 4.27 ± 0.41%, protein 5.23 ± 0.62%, fat 5.59 ± 2.65%, lactose 4.86 ± 0.22%, solids-not-fat (SNF) 11.45 ± 0.53%, urea 0.03 ± 0.01 μM, and citric acid 0.22 ± 0.04 °T. These values fall within the ranges previously reported for yak milk, where protein content typically ranges from 4.0 to 5.9%, fat from 5.5 to 7.5%, lactose from 4.0 to 5.9%, and SNF between 8 and 12% [2,8]. For example, Maiwa yak milk has been reported to contain approximately 3.7–4.1% protein and 6.2–7.7% fat, with total casein concentrations averaging 37–40 g/L (approximately 3.7–4.0%), which is consistent with the casein values observed in our study [22,23]. Furthermore, nitrogen distribution studies have shown that the total nitrogen in yak milk is approximately 0.8–0.9%, with non-protein nitrogen (including urea) accounting for a small proportion. The urea concentration identified in our study (~0.03 μM) is consistent with this expectation [21]. Similarly, citric acid—an important intermediate in energy metabolism during lactation—has been previously reported at elevated concentrations in yak milk, in line with the value we observed (~0.22 °T) [3,24]. Taken together, these data confirm that the eight traits measured in our study conform to the characteristic compositional profile of yak milk. Importantly, the precision (mean ± SD) and distribution of our data support robust comparison with existing benchmarks, establishing a solid foundation for subsequent genotype-related analyses (Table S1).

Although the milk yield of yaks is only a small fraction of that of ordinary dairy cows and limited to the lactation period, its superior nutritional density, including higher protein, fat, essential amino acids, minerals (such as calcium and iron), vitamin, and bioactive compounds with antibacterial and antioxidant properties contents, helps offset this limitation and enhances its overall value [2,3]. Yak milk contains approximately 40 g/L of caseins—about 1.5 times more than cow’s milk—and about 60 % of its total protein is casein, with β-casein accounting for over 45 % of this fraction [22]. Such a high β-casein content promotes softer curds and easier digestion, making it traditionally used in diluted form for infant feeding among Tibetan herders. Whey proteins—α-lactalbumin, β-lactoglobulin, serum albumin, lactoferrin, and immunoglobulins—are also significantly more abundant in yak milk than in bovine milk, contributing immunomodulatory, antimicrobial, and antioxidant effects [3]. Yak milk is further distinguished by higher concentrations of lactoferrin (~30 % above cow’s milk) and osteopontin (nearly double). Its lipid composition—comprising triglycerides, diacylglycerols, and polar lipids—differs significantly from human milk, with similar PUFA profiles and larger fat globules, suggesting strong potential for infant formula applications [4]. Yak milk is also richer in minerals like calcium (~1267 mg/L), phosphorus, magnesium, and essential trace elements (iron, zinc, selenium) compared to cow’s milk [5]. In sum, despite its limited availability, yak milk’s dense profile of casein, whey proteins, bioactive components, beneficial lipids, and minerals underscores its status as a nutrient-dense “superfood” that is particularly suited for infants, the elderly, immunocompromised individuals, and artisanal dairy production.

The analysis of two SNP loci within the *ATF6* gene in yaks—g.3_9812652G>T and g.3_9900243T>C—revealed distinct genetic and evolutionary patterns. At the g.3_9812652G>T locus, the G allele was found at a dominant frequency of 0.921, and genotype distributions conformed to Hardy–Weinberg equilibrium (*p* > 0.05), indicating a stable genetic structure and suggesting that purifying selection has favored this allele over time, leading to low polymorphism (PIC = 0.135). This is comparable to similar findings in other milk-quality genes in Gannan yaks, such as *PRKG1* and *LAP3*, which also exhibit low-to-moderate polymorphism while remaining useful in association studies [9,25]. In contrast, the g.3_9900243T>C locus showed a dominant C allele (frequency = 0.827) and moderate polymorphism (PIC = 0.245) but significantly deviated from Hardy–Weinberg equilibrium (*p* = 0.0312). According to population genetics theory, such a deviation may result from selection pressure, non-random mating, or genetic drift [26]. Considering *ATF6*’s well-established role in the endoplasmic reticulum stress response and its involvement in the differentiation and secretory function of mammary epithelial cells, particularly during lactation [13,27], it is plausible that artificial selection for milk production traits has influenced this SNP. Therefore, the g.3_9900243T>C locus emerges as a promising candidate for marker-assisted selection in yak breeding, especially given its deviation from equilibrium and moderate PIC, which suggest it may be functionally linked to milk quality attributes.

In this study, we assessed two intronic SNPs within the ATF6 gene—g.3_9812652G>T and g.3_9900243T>C—and their association with milk composition traits in yaks. GT individuals at g.3_9812652G>T exhibited significantly higher levels of casein, protein, acidity, and SNF compared to GG homozygotes (*p* < 0.01), whereas CT individuals at g.3_9900243T>C showed elevated casein, protein, SNF, and citric acid relative to CC homozygotes (*p* < 0.05). These effects persisted after adjustment for lactation number, highlighting strong associations with major protein-related traits. ATF6 encodes a transcription factor that plays a central role in the endoplasmic reticulum (ER) unfolded-protein response (UPR), which is activated under ER stress to restore protein-folding homeostasis [28,29]. In bovine mammary gland tissue, expression of ATF6 and related UPR components becomes elevated during early lactation, when the demand for protein and fat synthesis increases dramatically [30]. This upregulation supports proper folding, quality control, and secretion of nascent milk proteins such as caseins and whey proteins.

Although our two candidate SNPs are located within intronic regions, their strong trait associations suggest they may influence *ATF6* gene expression or splicing efficiency, potentially modulating protein synthesis and secretion capacity in lactating mammary cells. The g.3_9812652G>T variant, in particular, displayed a broader and more robust effect across multiple protein-related traits (casein, protein, and SNF), suggesting it may influence ATF6-mediated UPR signaling more significantly than the g.3_9900243T>C variant. Emerging evidence also links the UPR network to lipid metabolism regulation in the mammary gland. Under the metabolic stress of lactation, UPR components such as *ATF6* are believed to coordinate synthesis of both the protein and lipid components of milk, possibly via downstream targets that support fatty acid synthesis and secretion [13]. Thus, our findings relating SNPs in *ATF6* to casein, protein, and SNF traits are biologically plausible within this broader molecular context. In summary, these intronic SNPs within *ATF6* emerge as promising genetic markers for the selection of improved milk protein composition in yaks. Moving forward, functional validation—such as allele-specific expression assays or splicing analyses—and expanded studies in diverse yak populations are needed to confirm causal mechanisms and refine their utility in marker-assisted selection (MAS) targeting enhanced milk quality.

## 5. Conclusions

In this study, two single nucleotide polymorphisms (SNPs) within the *ATF6* gene—g.3_9812652G>T and g.3_9900243C>T—were identified in yaks and found to be significantly associated with several milk-quality traits, including protein, casein, fat, SNF, and lactose content. At the g.3_9812652G>T locus, significant differences between genotypes were observed for several milk quality traits, suggesting that this SNP may have potential practical value in selective breeding. These findings suggest that *ATF6* may be involved in the regulation of milk composition traits, though the underlying molecular mechanisms require further investigation. Overall, the results provide useful candidate genetic markers for future marker-assisted selection (MAS) programs aimed at improving yak milk production and quality.

## Figures and Tables

**Table 1:** Variation information and diversity parameters of ATF6 gene locus.

SNPs	Position	Genotypic Frequencies			Allelic Frequencies		PIC	He	Ne	HW *p* Value
g. 3_9812652G>T	Intron	GG	GT	TT	G	T	0.135	0.145	1.17	0.3380
		0.853	0.137	0.010	0.921	0.079				
g. 3_9900243T>C	Intron	CC	CT	TT	C	T	0.245	0.286	1.40	0.0312
		0.702	0.250	0.048	0.827	0.173				

Note: Heterozygosity (He), effective number of alleles (Ne), and polymorphism information content (PIC) were analyzed for the SNPs within the ATF6 gene. PIC values categorize polymorphism as follows: PIC < 0.25 indicates low polymorphism, 0.25 < PIC < 0.5 represents moderate polymorphism, and PIC > 0.5 denotes high polymorphism. A *p*-value > 0.05 suggests that the population is in Hardy–Weinberg equilibrium, indicating that the sample originates from a single Mendelian population. The genotypes GG, CGGT, CT, CC, and TT correspond to the SNP genotypes within the ATF6 gene, while C and T represent the alleles at these SNP loci.

**Table 2:** Correlation analysis of g.3_9812652G>T and g.3_9900243T>C genotypes with dairy quality traits in yak.

g. 3_9812652G>T								
Genotype	Casein/%	Protein/%	Acidity/°T	SNF/%	Lactose/%	Urea/μM	CitrAcid/°T	Fat/%
GG	4.23 ± 0.025	5.18 ± 0.039	12.91 ± 0.106	11.40 ± 0.033	4.90 ± 0.014	0.03 ± 0.001	0.22 ± 0.002	5.47 ± 0.165
GT	4.53 ± 0.063	5.58 ± 0.096	13.81 ± 0.264	11.74 ± 0.083	4.86 ± 0.034	0.03 ± 0.002	0.22 ± 0.006	6.01 ± 0.412
*p*-value	<0.01	<0.01	<0.01	<0.01	0.293	0.290	0.298	0.230
g. 3_9900243T > C								
Genotype	Casein/%	Protein/%	Acidity/°T	SNF/%	Lactose/%	Urea/μM	CitrAcid/°T	Fat/%
CC	4.21 ± 0.026	5.15 ± 0.040	12.85 ± 0.108	11.39 ± 0.036	4.90 ± 0.014	0.03 ± 0.001	0.23 ± 0.003	5.51 ± 0.180
CT	4.34 ± 0.043	5.32 ± 0.067	13.24 ± 0.183	11.54 ± 0.060	4.92 ± 0.024	0.03 ± 0.001	0.21 ± 0.004	5.44 ± 0.304
*p*-value	<0.01	0.027	0.070	0.031	0.516	0.728	0.017	0.855

Note: *p*-values derived from the general linear model (GLM) including both genotype and lactation number as fixed factors. Data are expressed as LSM ± SE. Means were adjusted for lactation number using GLM. Significance levels: *p* < 0.05.

## Data Availability

The original contributions presented in the study are included in the article; further inquiries can be directed to the corresponding authors.

## Supplementary Materials

The following supporting information can be downloaded at: https://www.mdpi.com/article/10.3390/ani15172524/s1. Table S1: The analysis of g.3_9812652G>T and g.3_9900243T>C genotypes with dairy quality traits in yak.
