# Supplementary material for: Genetic Variants in the ATF6 Gene and Their Relationship with Milk-Quality Traits in Yaks
Source: Animals (Basel). 2025 Aug 27;15(17):2524. doi: 10.3390/ani15172524 (PMC12427389; doi:10.3390/ani15172524)
Supplement: Supplementary file 1 [file animals-15-02524-s001.zip › animals-3772607-supplementary.pdf]

Table S1. The analysis of g.3_9812652G > T and g.3_9900243T > C genotypes with dairy quality traits in yak.

g. 3_9812652G > T								
Genotype	Casein/%	Protein/%	Acidity/°T	SNF/%	Lactose/%	Urea/ μ M	CitrAcid/°T	Fat/%
GG	4.23±0.37	5.18±0.56	12.90±1.51	11.40±0.48	4.90±0.19	0.03±0.01	0.22±0.04	5.47±2.49
GT	4.53±0.56	5.58±0.84	13.82±2.42	11.74±0.72	4.86±0.32	0.03±0.02	0.21±0.04	6.00±3.16
TT	4.80±0.43	5.83±0.57	14.19±0.82	11.46±0.29	4.56±0.19	0.02±0.01	0.19±0.03	10.54±4.02
Total	4.27±0.41	5.23±0.62	13.04±1.69	11.45±0.53	4.86±0.22	0.03±0.01	0.22±0.04	5.59±2.65

g. 3_9900243T > C								
Genotype	Casein/%	Protein/%	Acidity/°T	SNF/%	Lactose/%	Urea/μM	CitrAcid/°T	Fat/%
CC	4.21±0.36	5.15±0.54	12.84±1.49	11.39±0.46	4.90±0.20	0.03±0.01	0.22±0.04	5.51±2.46
CT	4.35±0.39	5.33±0.64	13.25±1.71	11.54±0.62	4.92±0.21	0.03±0.02	0.21±0.04	5.44±2.86
TT	4.84±0.75	6.03±1.03	14.84±2.92	11.88±0.76	4.61±0.35	0.02±0.01	0.21±0.03	7.72±3.53
Total	4.27±0.41	5.23±0.62	13.04±1.69	11.45±0.53	4.86±0.22	0.03±0.01	0.22±0.04	5.59±2.65

Note:Data are presented as mean ± standard deviation for each genotype. The "Total" column represents the overall mean ± standard deviation calculated from all 291 samples for each trait.
